# Supplementary material for: Actively addressed single pixel full-colour plasmonic display
Source: Nat Commun. 2017 May 10;8:15209. doi: 10.1038/ncomms15209 (PMC5436230; doi:10.1038/ncomms15209)
Supplement: Supplementary Information — Supplementary figures and supplementary notes. [file ncomms15209-s1.pdf]

Supplementary Note 1: Watershed-Based Grain Size Determination

Matlab's inbuilt watershed algorithm finds and labels regions surrounded by lines of local minima, or "watershed ridge lines", of arbitrary matrices. We use this generalized method on SEM images to identify grains of an aluminum film and their size. In order to effectively use the function, several image processing steps must be completed with care so that they do not influence the results of the grain size determination.

The method is as follows:

1) Define a pixel-to-nm length conversion factor.
2) Normalize possibly uneven background through Top-hat filtering
3) Optional 2D interpolation (will impact pixel to length conversion)
4) Gaussian Filter to eliminate static noise (filter must be much smaller than grain size but larger than static noise of image)
5) Find complimentary image
6) Suppress all image minima less than the noise floor of the image.
7) Apply watershed algorithm to find local regions surrounded by lines of local minima and their size.
8) Superimpose watershed result on image to verify output of function
9) Find histogram of watershed results which gives list of local regions with their respective number of pixels
10) Convert number of pixels into an area with pixel-to-nm conversion factor.
11) As grains are of random shapes, assume grains are spheroidal and obtain diameter of respective circle given area.
12) Gaussian fit the resulting histogram plot

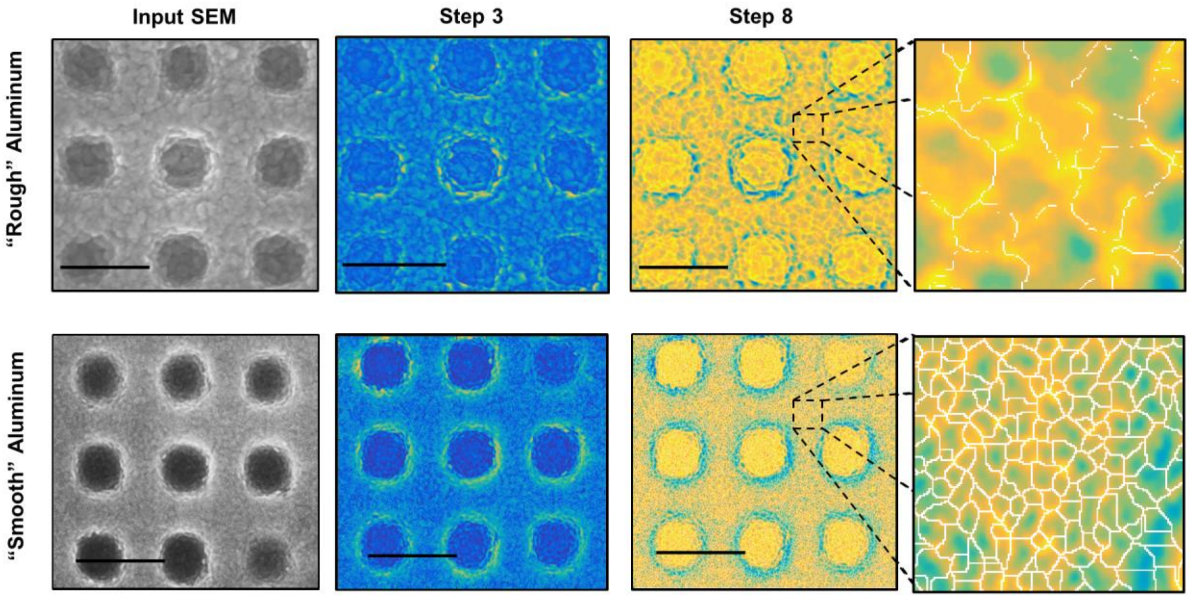

Supplementary Figure 1 | Watershed-Based Grain Size Determination. The input SEM images for the "rough" and "smooth" aluminum thin film are shown at various steps in the watershed method. The method results in a list of regions with the number of pixels they contain. The area of each region is then converted into an approximate grain diameter. Regions are mapped back on the image in the form of white lines to confirm the methods functionality. Scale bars are 300 nm.

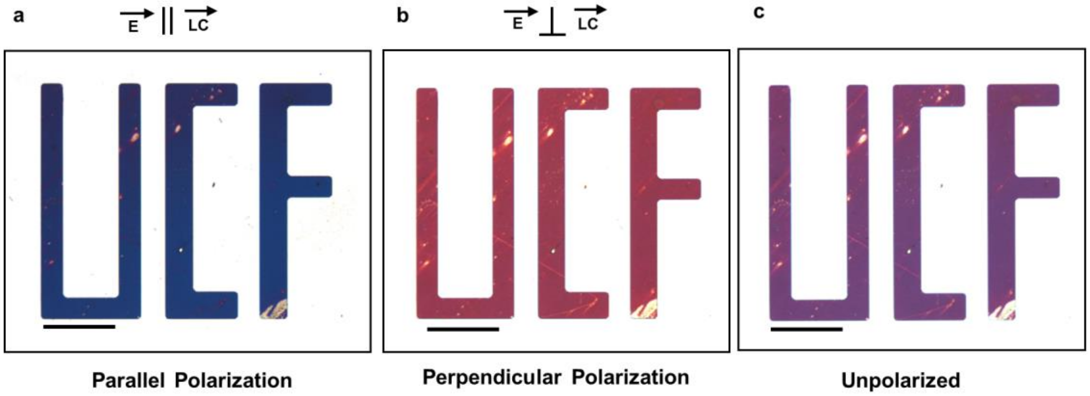

Supplementary Figure 2 | Device Under Unpolarized Light. The off-state behavior of the surface results in a blue or red when the electric field of incident light is **a)** parallel or **b)** perpendicular to the top LC alignment director, respectively. Without a polarizer, the device reflects a superposition of the two orthogonal polarization colors, resulting in **c)** a purple. Scale bars are 150 μm.

Supplementary Note 2: LC Modeling and Jones Matrix Method

The bulk LC dynamics are simulated using the TechWiz LCD 3D (Sanayi) software package. The Finite Element Method (FEM) solver finds the minimum energy state of the LC director given LC material parameters, boundary conditions and applied voltages. A unit cell of the simulated nano-well array can be seen in SI Fig. 3 (a) where the LC layer is approximated with 50 layers which has been found to give well converged results. The simulations are run in increments of 0.1 V and result in LC director tensors that represent the LC orientation throughout the cell. We then convert this data into the “tilt” (θ) and “twist” (φ) angles of the LC which can be seen in SI Fig. 3(b). These simulation results will then be used in a Jones Matrix formulation to find the optical behavior of the LC cell.

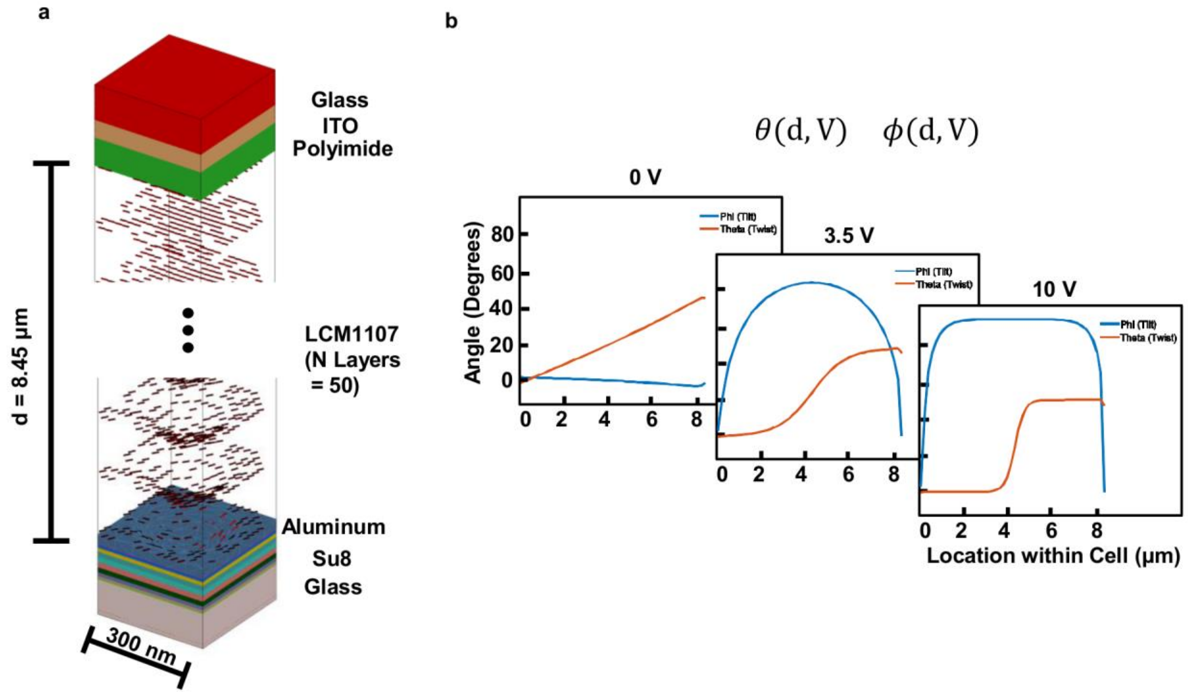

Supplementary Figure 3 | FEM LC Modeling and Results. A unit cell of the simulated nano-well array can be seen in **a)**, consisting of 50 layers of LC. The cell gap is 8.45 µm and is obtained through experiment. The top polyimide has as strong anchoring condition (1e^{-4} Nm^{-1}) with a pre-tilt of 3° and in-plane director of 0°. The aluminum surface is also simulated with a strong anchoring and in-plane director of 45° which is derived from our previous work. **b)** shows the resulting θ and ϕ as a function of cell position and voltage.

Once the direction of the LC throughout the cell is known, we use a Jones Matrix formulation to solve for the cell’s optical properties. This is done by approximating the LC layer as a stack of N uniaxial crystals and combining their transfer matrices,

$$LC_{\mathrm{j}} = \prod_{\mathrm{j}=1}^{N} M_{\mathrm{j}} \tag{1}$$

where

$$M_{\mathrm{j}} = Rot(-\varphi) * \begin{bmatrix} e^{-i\frac{g_{\mathrm{j}}}{2}} & 0 \\ 0 & e^{i\frac{g_{\mathrm{j}}}{2}} \end{bmatrix} Rot(\varphi) \quad . \tag{2}$$

Here, Rot is the rotation matrix and φ is the twist angle of the LC for a given layer j.

$$Rot(\varphi) = \begin{bmatrix} \cos(\varphi) & \sin(\varphi) \\ -\sin(\varphi) & \cos(\varphi) \end{bmatrix} \tag{3}$$

and

$$g_{\mathrm{j}} = \frac{2\pi}{\lambda} \Delta n_{\mathrm{j}} d_{\mathrm{j}} \quad . \tag{4}$$

This phase term depends on the thickness, d_{j}, and birefringence, Δn_{j}, of each individual layer j. The birefringence is given by

$$\Delta n_{\mathrm{j}} = n_{\mathrm{e}}(\theta_{\mathrm{j}}) - n_{\mathrm{o}} \quad , \tag{5}$$

where $n_e(\theta_j)$ is the effective extraordinary index of the LC given a tilt angle of θ_i

$$\frac{1}{n_{\mathrm{e}}^2(\theta)} = \frac{\cos^2(\theta)}{n_{\mathrm{e}}^2} + \frac{\cos^2(\theta)}{n_{\mathrm{o}}^2} \quad . \tag{6}$$

Here, n_e and n_O are the extraordinary and ordinary indices of the liquid crystal.

To find the strength and phase of light at each layer, we iteratively perform the above matrix multiplication for incident light that is parallel to the top LC alignment director and which we define in the x-direction.

$$\begin{bmatrix} E_{\mathrm{x}} \\ E_{\mathrm{y}} \end{bmatrix}_{\mathrm{j}} = LC_{\mathrm{j}} \begin{bmatrix} 1 \\ 0 \end{bmatrix} \tag{7}$$

We then use this to find the E-field that excites the plasmonic surface. To determine the amount of each orthogonal mode of the surface that's excited, we project the exciting light on the states of the surface which are defined by the in-plane angle of the LC atop it, $\theta_N \sim = \frac{\pi}{4}$.

$$\alpha = |\langle \psi_1 | LC_N | E_{\mathrm{in}} \rangle| \tag{8}$$
$$\beta = |\langle \psi_2 | LC_N | E_{\mathrm{in}} \rangle| \tag{9}$$

This results in wavelength and voltage dependent weighting factors, α and β, such that the reflection spectra of the surface in the low voltage regime, where bulk LC deforms but remains anchored LC on aluminum surface, is a superposition of the two orthogonal off-state modes.

$$|\psi_{\mathrm{V}}\rangle = \alpha|\psi_1\rangle + \beta|\psi_2\rangle \tag{10}$$

These weighting terms also satisfy the following condition.

$$\alpha^2 + \beta^2 = 1 \tag{11}$$

The resulting α and β are shown in SI Fig. 4, respectively. Here we can see that an effective flipping occurs within the 3.5 V to 4 V region. These weighting values are used on the two orthogonal off-state reflection spectra of the surface to closely match experimental measured spectra at this flipping region, as seen in Fig. 3 (e). While in Fig. 4 (a), these weighting terms are

applied to the FDTD simulated reflection spectra for the surface's two orthogonal off-state modes.

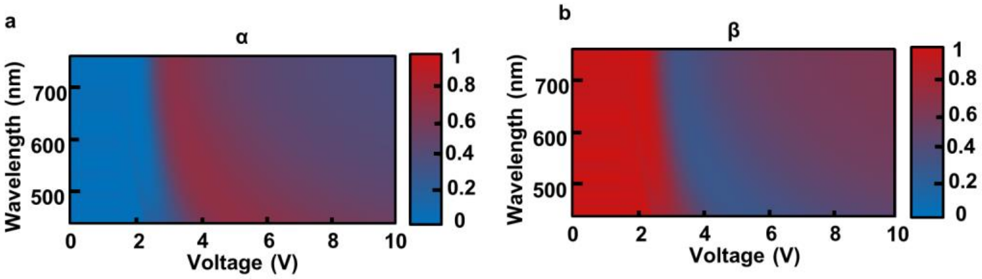

Supplementary Figure 4 | Wavelength and Voltage Dependent Weighting Factors. (a) *α* and **(b)** *β*

To complete the model and find the reflection out of the device, we must consider reflection, a reverse pass through the LC and exit through the polarizer. It is possible to incorporate these into the matrix method by using the transfer matrix method in reverse, or by using the following which is based on the symmetry of the system.

$$R = \left| [cos\varphi_1 \quad sin\varphi_1] H M H^{-1} M \begin{bmatrix} cos\varphi_1 \\ sin\varphi_1 \end{bmatrix} \right|^2 \tag{12}$$

Where is given by.

$$H = \begin{bmatrix} \cos\varphi_N & \sin\varphi_N \\ \sin\varphi_N & -\cos\varphi_N \end{bmatrix} \tag{13}$$

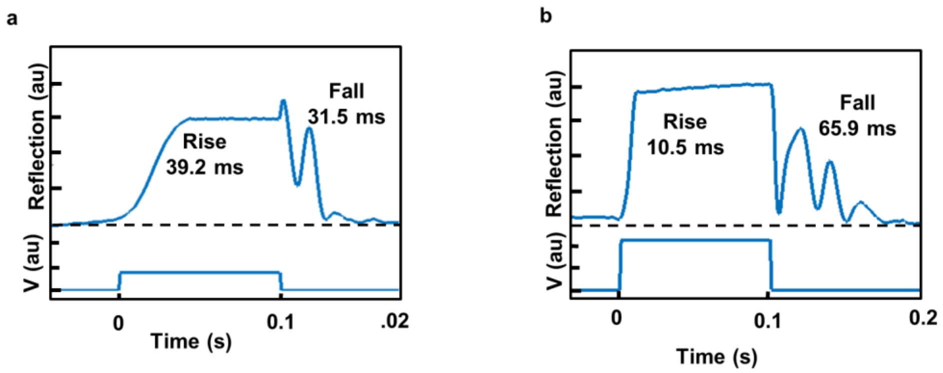

Supplementary Figure 5 | Response time measurements. Using the experimental setup outlined in Figure 3 (b) (633 nm He-Ne laser, polarizing beam splitter and photodiode) measurements of response time are shown for (**a**) 2.6 V and (**b**) 5.4 V (1kHz AC). These give total switching times of 70.7 ms and 76.4 ms, respectively. Cell gaps are 8.45 µm.
